# Isolated Radial Nerve Palsy as a Complication After Anterior Dislocation of the Glenohumeral Joint: A Case Report and Clinical Review

**DOI:** 10.1177/2324709619844289

**Published:** 2019-05-14

**Authors:** Grigorios Kastanis, Petros Kapsetakis, George Velivasakis, Manolis Spyrantis, Anna Pantouvaki

**Affiliations:** 1 General Hospital of Heraklion-Venizeleio, Crete, Greece

**Keywords:** radial palsy, anterior shoulder dislocation, terrible triad of the shoulder

## Abstract

Shoulder dislocation is the most common feature in emergencies, while the anterior dislocation of the glenohumeral joint is the most frequent and requires reduction. Accompanied nerve injury is common with an incidence of 21%, while radial nerve palsy is very rare. We describe the case of a 56-year-old man who presented with an anterior dislocation of the left shoulder due to a fall on an outstretched hand with wrist drop 8 hours after injury. Neurological examination revealed loss of sensation along the radial border of the forearm. Closed reduction with Kocher procedure was performed. Magnetic resonance image demonstrated a rotator cuff tear, and 3 weeks after the injury electromyography showed complete radial nerve palsy. A physiotherapy program was applied to the wrist and fingers with the goal of maintaining a full passive range of motion in all joints affected while shoulder rehabilitation started 6 weeks after his fall. Isolated radial nerve palsy associated with an anterior dislocation of the shoulder is very rare but not impossible to occur. Correct diagnosis of the nerve injury associated with the anterior dislocation is very important because it has serious implications on the management and activity morbidity.

## Introduction

Shoulder dislocation accounts for 50% of all dislocated joints, while anterior dislocation in 95% of cases require reduction by emergency physicians. The frequency of brachial plexus injury associated with acute anterior shoulder dislocation is very common (18% to 86% of patients in different studies having brachial plexus injury after shoulder dislocation with a simple fall), with an incidence based on clinical diagnosis from 3% to 21%, while using electromyography (EMG) the incidence arises to 9% to 65%.^1-3^

Isolated axillary nerve is most frequently involved because of its close association with the glenohumeral joint,^3^ but isolated radial palsy after low-energy shoulder dislocation is not clearly defined. Visser et al^3^ determined the incidence in 7%, while Deldet et al^4^ determined the incidence in 18%. In this article, we describe the case of a 56-year-old man who had a fall on an outstretched hand from his standing height and appeared to have a terrible triad of symptoms, an anterior shoulder dislocation, a peripheral nerve injury, and a rotator cuff tear.

The main aim of this study is to increase the index of clinical suspicion for emergency physicians, of the presence of an isolated radial nerve palsy after anterior dislocation of the shoulder, because delay in recognition and following treatment can have long-term impact on patients’ functional outcome.^5,6^

## Case Report

**Figure 1.**
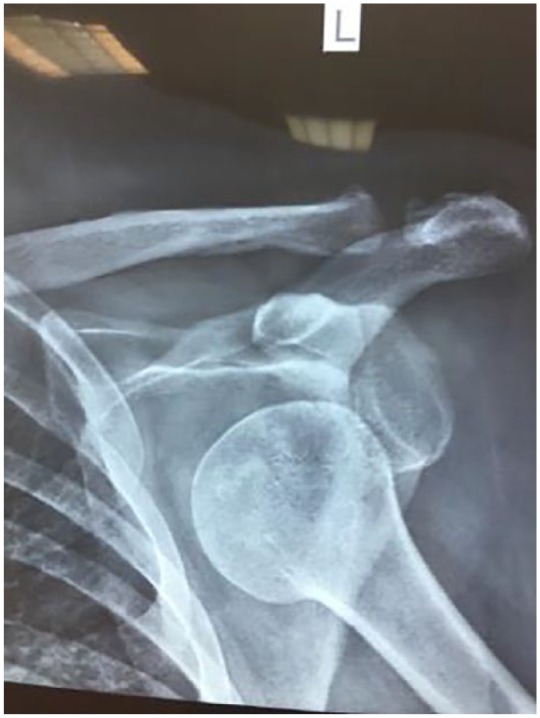
AP X-ray of the shoulder illustrating anterior shoulder dislocation.

**Figure 2.**
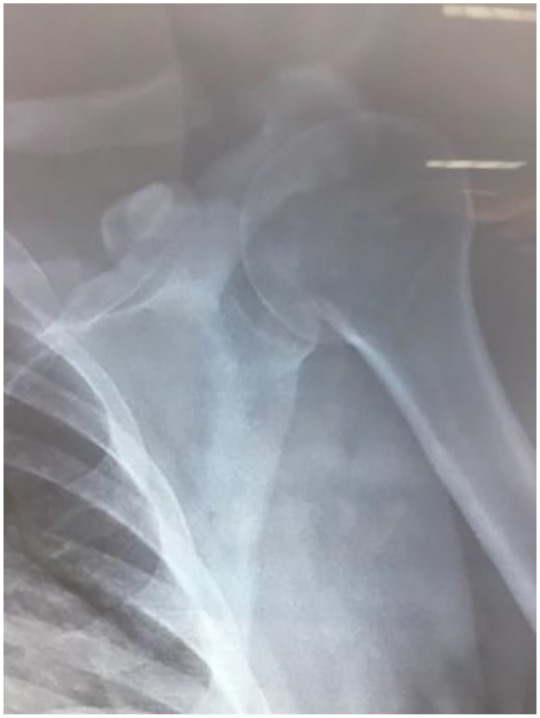
Post-reduction X-ray with restoration of the normal position of the humeral head in the glenoid.

**Figure 3.**
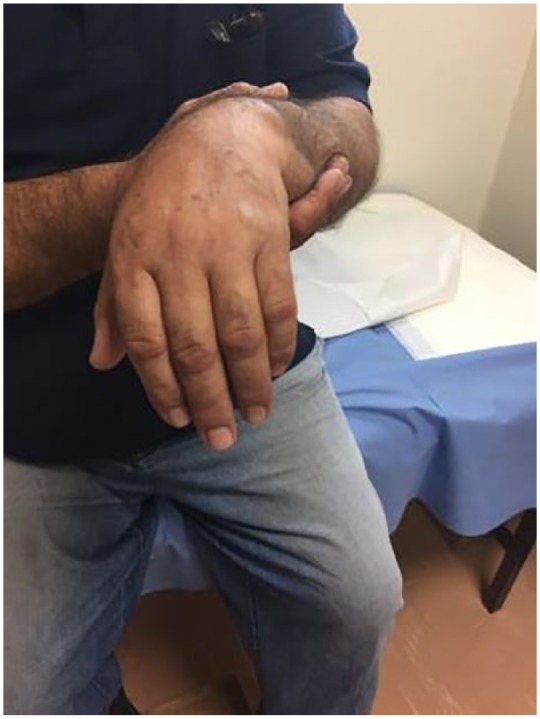
Photo illustrating drop hand of the patient when arrived in the emergency department.

**Figure 4.**
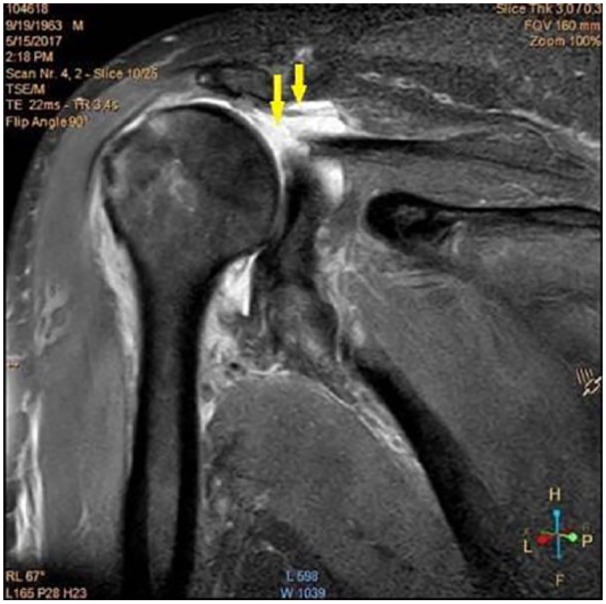
Magnetic resonance image of the shoulder demonstrates a rupture of the rotator cuff.

A 56-year-old man was presented to the emergency department 8 hours after a fall from his own height with pain, bruising, and inability to move his arm. On clinical examination he had loss of the normal contour of the deltoid and the acromion was prominent posteriorly and laterally. Ηe was holding his injured arm but he could not extend the elbow, the wrist (wrist drop) actively, and fingers from neutral position. The neurological examination that followed revealed numbness along the radial border of the forearm in the distribution of the radial sensory nerve. Median and ulnar nerve were intact and peripheral pulses were present. A plain AP X-ray and axillary view confirmed the diagnosis of anterior shoulder dislocation (Figure 1). The dislocation was reducted under sedation using Kocher technique and post-reduction radiographs affirmed the right position of the humeral head in the glenoid, but sensory paresthesia and drop hand remained (Figures 2 and 3). The shoulder was immobilized in an arm sling, a wrist splint was applied, and the patient was discharged taking instructions. In the follow-up, a few days later a shoulder magnetic resonance imaging (MRI) revealed a rotator cuff tear (Figure 4), and 3 weeks after the injury, EMG and nerve conduction studies showed no response in latency and conduction velocities (1.6 ms, 4.7 mV) in the radial motor nerve distribution, which indicated a complete radial nerve palsy. The patient started physiotherapy for the wrist and the fingers with the goal to maintain a full passive range of motion in all joints. Patient performed passive, assistive, and self-assistive movements and stretches to wrist maintaining the ROM (range of motion). Passive movements using continuous passive motion equipment were used to reduce stiffness and pain due to edema and inability to straighten the fingers. To regain muscle strength the patient was encouraged to do hand and finger exercises using physiotherapy tools such as silicone relief pressure hand grip and gripper resistance bands. A shoulder rehabilitation program started 6 weeks after his fall with passive, assistive movements to increase ROM and to reduce stiffness and pain in the joint. An exercise regime was given to regain gradually the strength and the neuromuscular control of the shoulder while education of proprioception was critical in returning safely to functional activities. The importance of early intervention following early diagnosis is to prevent muscle atrophy, the development of secondary deformities, and to maintain adequate muscle trophism during re-innervation.

## Discussion

Glenohumeral joint has the greatest range of motion of any other joint in the body. Due to the uniqueness of the joint’s design, shoulder is the most frequently dislocated major joint accounting to up to 45% of dislocations.^7^

Over 95% of these dislocations are anterior, while posterior and luxatio erecta (inferior shoulder dislocation) occur rarely. High-energy injuries are the cause in the young males, although in older patients it is more frequent in women after low-energy injuries.^8^ Various spectrums of complications are associated with these injuries that increase the rate of recurrence, particularly to patients younger than 20 years, whereas the incidence can be high as 80% to 92%.^9,10^ Bankart (an injury of the anterior-inferior glenoid labrum of the shoulder) and Hill Sachs (a cortical depression in the posterolateral head of the humerus) lesions are essential for the future risk of recurrent dislocation. Greater tuberosity fractures can also coexist but are common in the older patients, while rotator cuff can be present in 14% to 43% of the cases.^8^

Vascular injuries are not so common after dislocations, but axillary artery rupture has been reported in the literature because of the inelasticity of these vessels in older patients and might be accompanied with brachial plexus injury with an incidence rate of 27% to 44%,^11^ as a cause of the compression of the hematoma formed. Axillary arterial thromboses have been also reported.^12^ Also reported is a case of an axillary pseudoaneurysm with a delayed neurovascular insult, compressing the brachial plexus, complicating a primary anterior shoulder dislocation.^13^

Nerves are vulnerable to injury from traction because the distance between the anchorage points of nerves in the upper limb is short especially for radial and musculocutaneous nerve. Axillary nerve is most frequently involved because it is relatively fixed as it travels through the quadrangular space and its course around the surgical neck of humerus. Neurologic examination is essential before and after reduction, and according to a study nearly 10% of the patients did not have a neurological examination performed in the emergency department.^14^ When injuries are not neurologically assessed, before physician’s manipulations, any nerve deficits could be easily attributed to iatrogenic injury.^15^ Visser et al^3^ found that 42% of their 77 patients had axillary nerve injury after low-energy shoulder dislocation.

These injuries are more common based on EMG studies.^3^ Axillary nerve injury is about 42%, following by suprascapular 14%, musculocutaneous 12%, ulnar 8%, radial 7%, and median nerve 4%. Hems et al^16^ reviewed 55 patients with anterior dislocation with lesions of the terminal branches of the infraclavicular brachial plexus and found that axillary and ulnar nerve were most frequently injured. Most of these injuries are temporary neuroapraxias and they can resolve within 6 to 12 months, but in the literature there are also reported cases of permanent axillary deficit.^17^

Radial nerve originates from posterior cord of the infraclavicular brachial plexus and provides the motor branches to the extensor muscles of the forearm and arm. Radial nerve palsy can occur after a fracture in the distal third of the humerus (Holstein Lewis fractures), when prolonged pressure occurs due to compression of the nerve (Saturday night palsy) or because of a big hematoma, but also iatrogenic injuries are not unusual after surgical treatment of various pathologies. Radial nerve palsy after glenohumeral dislocation occurs rarely in the literature. Al-Khateeb et al reported a 74-year-old female with drop wrist, anterior shoulder dislocation following low-energy trauma,^18^ while Johnson^19^ described isolated radial nerve palsy in a 86-year-ole man with anterior shoulder dislocation. Yeap et al,^15^ in a study of 86 patients who were examined with shoulder dislocation, found that 8 of them had sustained neurological injuries and there was one case with isolated radial nerve palsy. Electrodiagnostic examination of 11 patients by Liverson with shoulder dislocation revealed 5 cases with injury of posterior cord (both axillary and radial nerve) not previously reported.^20^

Anterior shoulder dislocation complicated with peripheral nerve injury and rotator cuff rupture was first described by Gonzalez and Lopez^21^ and some years later named unhappy triad and then terrible triad of the shoulder. A combination of nerve injury and an MRI-proven cuff rupture was found in 7 of 77 patients studied by Visser et al.^3^ The diagnosis of the rotator cuff tear is very difficult after a reduction in the emergency because of the pain and swelling, but the inability to abduct the shoulder is a sign of rotator cuff rupture, brachial nerve palsy, or both.^22^ Berbig et al^23^ recommended ultrasound examination in patients over 40 years with shoulder dislocation, and they found a correlation between full thickness tears and inability to abduct the arm 2 weeks after dislocation. In our case, MRI was performed 5 days after the injury in the follow-up, because of the suspicion that arose from the clinical examination. Early diagnosis and surgical repair is related with better outcomes and avoidance of fatty degeneration and atrophy of the rotator cuff muscles.^22^ Our patient had almost full thickness tear of the supraspinatus tendon and partial tear of infraspinatus tendon, but he preferred the conservative way of treatment.

EMG studies are proposed 3 weeks after injury associated with palsies or paralyses of the upper limb.^3^ It is not useful after a shoulder reduction because the characteristic degeneration of the muscle surface membrane does not occur immediately after the nerve injury and cannot be detected by needle electrode examination for approximately 2 to 3 weeks. These studies can help to note the nerve injury, can be used as a baseline for future comparison (EMG examination), and are extremely valid especially in patients with massive rotator cuff tears when coexistent nerve palsy is skipped in the diagnosis.^24^ Nerve injuries following shoulder dislocation are neuropraxic or axonotmetic lesions with good recovery and they have to be observed for spontaneous recovery for several months. A neuroapraxia should demonstrate an almost full recovery by 3 months, while axonotmetic injuries should demonstrate re-innervation with an expected recovery by 6 to 7 months.^15,25,26^ If they do not demonstrate clinical (Tinel test, MRC scale at least grade 3) or electrical recovery (nerve conduction and EMG studies) after 3 to 6 months (relative indication for surgical intervention) surgical intervention should be taken into consideration. Sensory recovery precedes motor recovery constituting the best indicator for recovery potential. These injuries may benefit from early exploration and neurolysis, and if on examination at 9 months there is no progress in recovery then rehabilitation is unlikely and nerve transfer may be a good option.^27^

## Conclusion

We report a case of a terrible triad with a radial palsy and a rotator cuff tear due to anterior shoulder dislocation, and only a few have been reported in the literature. This injury is very rare and for this reason a high index of suspicion is necessary for all the emergency physicians especially when the patients are geriatric and the level of communication is difficult. Correct diagnosis of the nerve injury associated with anterior dislocation is very important because of serious implications on management and activity morbidity.

## References

[1] Blom S Dahlback LO. Nerve injuries in dislocations of the shoulder joint and fractures of the neck of the humerus. A clinical and electromyographical study. Acta Chir Scand. 1970;136:461-466. 5518118

[2] Toolanen G Hildingsson C Hedlund T Knibestol M Oberg L. Early complications after anterior dislocation of the shoulder in patients over 40 years. An ultrasonographic and electromyographic study. Acta Orthop Scand. 1993;64:549-552. 8237322 10.3109/17453679308993690

[3] Visser CP Coene LN Brand R Tavy DL. The incidence of nerve injury in anterior dislocation of the shoulder and its influence on functional recovery. A prospective clinical and EMG study. J Bone Joint Surg. 1999;81:679-685. 10.1302/0301-620x.81b4.9005 10463745

[4] Deldet P Cauchoix A. Les paralysies dans les luxations de l’epaule. Rev Chir. 1910;41:327.

[5] Beeson MS. Complications of shoulder dislocation. Am J Emerg Med. 1999;17:288-295. 10337892 10.1016/s0735-6757(99)90127-4

[6] Janitzky AA Akyol C Kesapli M Gungor F Imak A Hakbilir O. Anterior dislocation in busy emergency departments: The external rotation without sedation and analgesia method may be the first choice for reduction. Medicine (Baltimore). 2015;(47):e1852. doi:10.1097/MD.0000000000001852 26632681 PMC5058950

[7] Rockwood CA Jr Wirth MA Subluxations and dislocations about the glenohumeral joint. In: CA Jr Rockwood DP Green RW Bucholz JD Heckman, eds. Fractures in Adults. Philadelphia, PA: Lippincott-Raven; 1996:1193-1341.

[8] Cutts S Prempeh M Drew S. Anterior shoulder dislocation. Ann R Coll Surg Engl. 2009;91:2-7. 19126329 10.1308/003588409X359123 PMC2752231

[9] Rowe CR. Prognosis in dislocations of the shoulder. J Bone Joint Surg. 1956:38-A:957-977. 13367074

[10] McLaughlin HL MacLellan DI Recurrent anterior dislocation of the shoulder. II. A comparative study. J Trauma. 1967;7:191-201. 6018942 10.1097/00005373-196703000-00002

[11] Graham JM Mattox KL Felican DV DeBakey MF. Vascular injuries of the axilla. Αnn Surg. 1982;195:232-238. 10.1097/00000658-198202000-00020 PMC1352449 7055402

[12] Popescu D Fernández-Valencia JA Combalía A. Axillary arterial thrombosis secondary to anterior shoulder dislocation. Acta Orthop Belg. 2006;72:637-640. 17152431

[13] McCann PA Barakat MJ Wand JS. Delayed brachial plexus compression secondary to anterior shoulder dislocation—the late consequence of an axillary artery pseudoaneurysm: a case report. Injury Extra. 2006;37:458-461.

[14] Duralde XA Neurologic injuries in the athletes shoulder. J Ath Train. 2000;35:316-328. PMC1323394 16558645

[15] Yeap JS Lee DJ Fazir M Kareem BA Yeap JK. Nerve injuries in anterior shoulder dislocations. Med J Malaysia. 2004;59:450-454. 15779576

[16] Hems TE Mahmood F. Injuries of the terminal branches of the infraclavicular brachial plexus: patterns of injury, management and outcome. J Bone Joint Surg Br. 2012;94:799-804. 22628595 10.1302/0301-620X.94B6.28286

[17] Galvin JW Eichinger JK. Outcomes following closed axillary nerve injury: a case report and review of the literature. Mil Med. 2016;181:e291-e297. 26926757 10.7205/MILMED-D-15-00205

[18] Al-Khateeb H Naser M Selvanayagam N Rahman A Basheer S. Isolated radial nerve injury following anterior shoulder dislocation: case report and literature review. Ortho Rheum Open Access J. 2017;7:555707. doi:10.19080/OROAJ.2017.07.555707

[19] Johnson JR. Radial nerve palsy after an anterior shoulder dislocation: a case report. PM R. 2011;10(suppl 1):239.

[20] Liveson JA. Nerve lesions associated with shoulder dislocation; an electrodiagnostic study of 11 cases. J. Neurol Neurosurg Psychiatry. 1984;47:742-744. 6747652 10.1136/jnnp.47.7.742 PMC1027907

[21] Gonzalez D Lopez R Concurrent rotator-cuff tear and brachial plexus palsy associated with anterior dislocation of the shoulder. A report of two cases. J Bone Joint Surg Am. 1991;73:620-621. 2013606

[22] Groh GI Rockwood CA Jr. The terrible triad: anterior dislocation of the shoulder with rupture of the rotator cuff and injury to the brachial plexus. J Shoulder Elbow Surg. 1995;4(1 pt 1):51-53. 7874565 10.1016/s1058-2746(10)80008-4

[23] Berbig R Weishaupt D Prim J Shahin O. Primary anterior shoulder dislocation and rotator cuff tears. J Shoulder Elbow Surg. 1999;8:220-225. 10389076 10.1016/s1058-2746(99)90132-5

[24] Simonich SD Wright TW. Terrible triad of the shoulder. J Shoulder Elbow Surg. 2003;12:566-568. 14671520 10.1016/s1058-2746(03)00216-7

[25] Kosiyatrakul A Jitprapaikulsarn S Durand S Oberlin C. Recovery of brachial plexus injury after shoulder dislocation. Injury. 2009;40:1327-1329. 19540487 10.1016/j.injury.2009.05.015

[26] Lee S Saetia K Saha S Kline DG Kim DH. Axillary nerve injury associated with sports. Neurosurg Focus. 2011;31:E10. doi:10.3171/2011.8.FOCUS11183 22044099

[27] Duncan A Power D. Axillary nerve injury associated with glenohumeral dislocation: a review and algorithm for management. EFORT Open Rev. 2018;3:70-77. 29657847 10.1302/2058-5241.3.170003 PMC5890131

